# Early Diagnosis and Surgical Treatment for Necrotizing Fasciitis: A Multicenter Study

**DOI:** 10.3389/fsurg.2017.00005

**Published:** 2017-02-07

**Authors:** Evangelos P. Misiakos, George Bagias, Iordanis Papadopoulos, Nickolaos Danias, Paul Patapis, Nickolaos Machairas, Theodore Karatzas, Nickolaos Arkadopoulos, Konstantinos Toutouzas, Nickolaos Alexakis, Manousos N. Konstantoulakis, George Zografos, Vasilis Smyrniotis, Gregory Kouraklis, Anastasios Machairas

**Affiliations:** ^1^3rd Department of Surgery, Medical School, National and Kapodistrian University of Athens, Attikon University Hospital, Athens, Greece; ^2^4th Department of Surgery, Medical School, National and Kapodistrian University of Athens, Attikon University Hospital, Athens, Greece; ^3^2nd Propedeutic Department of Surgery, Medical School, National and Kapodistrian University of Athens, Laikon General Hospital, Athens, Greece; ^4^1st Propedeutic Department of Surgery, Medical School, National and Kapodistrian University of Athens, Hippokration General Hospital, Athens, Greece

**Keywords:** necrotizing fasciitis, LRINEC score, debridement, VAC therapy, Fournier’s gangrene

## Abstract

**Background:**

Necrotizing fasciitis (NF) is a group of relatively rare infections, usually caused by two or more pathogens. It affects the skin and subcutaneous tissues of lower and upper limbs, perineal area (Fournier’s gangrene), and the abdominal wall. Early diagnosis and aggressive surgical management are of high significance for the management of this potentially lethal disease.

**Methods:**

We conducted a retrospective study in patients who presented, during the last decade, at four University Surgical Departments in the area of Athens, Greece, with an admission diagnosis of NF. Demographic, clinical, and laboratory data were gathered, and the preoperative and surgical treatment, as well as the postoperative treatment was analyzed for these patients.

**Results:**

A total of 62 patients were included in the study. The mean age of patients was 63.7 (47 male patients). Advanced age (over 65 years) (*P* < 0.01) and female sex (*P* = 0.04) correlated significantly with mortality. Perineum was the mostly infected site (46.8%), followed by the lower limbs (35.5%), the upper limbs, and the axillary region (8.1%). Diabetes mellitus was the most common coexisting disease (40.3%), followed by hypertension (25.8%) and obesity (17.7%). The most common symptom was local pain and tenderness (90.3%). Septic shock occurred in eight patients (12.9%) and strongly correlated with mortality (*P* < 0.01). Laboratory data were used to calculate the LRINEC score of every patient retrospectively; 26 patients (41.9%) had LRINEC score under 6, 20 patients (32.3%) had LRINEC score 6–8, and 16 patients (25.8%) had LRINEC score >9. Surgical debridement was performed in all patients (mean number of repeated debridement 4.8), and in 16 cases (25.8%) the infected limb was amputated. The mean length of hospital stay was 19.7 days, and the overall mortality rate of our series was 17.7%.

**Conclusion:**

Diagnosis of NF requires high suspect among clinicians, as its clinical image is non-specific. Laboratory tests can depict the severity of the disease; therefore, they must be carefully evaluated. Urgent surgical debridement is the mainstay of treatment in all patients; the need of repetitive surgical debridement is undisputed.

## Introduction

Necrotizing fasciitis (NF) is actually a group of relatively uncommon, but life-threatening infections, which have the same clinical course and require urgent treatment. It usually affects the skin, soft tissues, and muscles, and may progress rapidly through the fascia planes, resulting in gradual destruction of the fascia. Its brisk clinical development can be explained by the pathogens that are usually involved, as in the majority of cases, a synergy of two or more pathogens are detected (1). NF is commonly located in the lower extremities, the perineum and genital area (Fournier’s gangrene), the abdominal wall, and in upper extremities (2). NF is sometimes falsely called as “gas gangrene” due to free gas accumulating in the soft tissue spaces, giving the characteristic image of gas gangrene on plain X-rays and computed tomography (CT) scans (3); however, this term describes only the third type of NF, where the infection is caused mainly by *Clostridium* species.

Necrotizing fasciitis clinical onset and outcome has been correlated with numerous comorbidities. The most frequent comorbidity is diabetes mellitus, which can be found in 40–60% of patients with any NF type (4). Currently, it is under consideration whether diabetes mellitus is correlated with worse outcome or not (5, 6). Obesity and preexisting hypertension are also present in patients with NF, with no correlation with higher mortality rates (7). Although advanced age is widely accepted as a prognostic factor for NF, it is currently uncertain whether sex may influence NF clinical course and outcome. Nevertheless, patients who are critically ill on admission may suffer from the fulminant form of NF.

Given its rapid progression, it is understandable that any delay in the diagnosis of NF could prove fatal. Although it is not easy, an early diagnosis must be obtained within 4 h after admission. During this limited time, surgeons must evaluate mainly laboratory results, which can be very helpful not only for establishing the diagnosis but also for estimating the severity of the infection (8). When NF is suspected, patients should be managed with aggressive fluid resuscitation, antibiotic treatment, and emergency surgical debridement. The extent, the number of consecutive surgical debridements, and other operations that may be needed are decisive factors for better outcome (9).

In this retrospective study, we analyzed the profile of patients with NF and evaluated the diagnostic modalities that we used to set an early diagnosis. Furthermore, we present our therapeutic strategy that had a clear effect in the clinical outcome.

## Patients and Methods

This is a retrospective study, including all patients admitted at our Departments (1st Propedeutic Department of Surgery, Hippokration General Hospital; 2nd Propedeutic Department of Surgery, Laikon General Hospital; 3rd Department of Surgery, Attikon University Hospital; and 4th Department of Surgery, Attikon University Hospital) between 2005 and 2015 with a diagnosis of NF. The initial diagnosis was established mainly at the Emergency Surgical Department, based on patient’s clinical data and laboratory results. In equivocal cases, the diagnosis was ascertained with imaging studies, mainly CT scan and/or plain radiography. The medical records of these patients were thoroughly analyzed. Specifically, we gathered the demographic data, the presenting symptoms (systematic and local changes of the infected skin), the site of infection, the possible comorbidities, and the results of the laboratory and imaging studies. Utilizing laboratory data, we retrospectively calculated the LRINEC score of every patient. The Laboratory Risk Indicator for NF was proposed by Wong et al., which consisted of six laboratory results (C-reactive protein, white-blood-cell count, hemoglobin, sodium, creatinine, and glucose) and stands for an early diagnosis and classification of patients into risk categories. In addition, LRINEC score can provide information about the severity of infection, which is helpful in comparing the results of the treatment. The cultures along with antibacterial sensibilities from the wound samples could not be safely recorded and therefore were not included in our study. We analyzed the preoperative management, the surgical procedure, and the postoperative treatment. Moreover, we evaluated the length of hospital stay among patients with different postoperative management and the overall mortality rate. Statistical analysis was made using SPSS v23.0 software. All values are presented as mean and/or median followed by the range. A standard Chi squared analysis was used for statistical correlations, and the level of statistical significance was set at *P* = 0.05. We also ran a univariate analysis for determining prognostic factors for mortality using a Cox regression model. The study has been approved by the local Ethics Committee.

## Results

**Figure 1:**
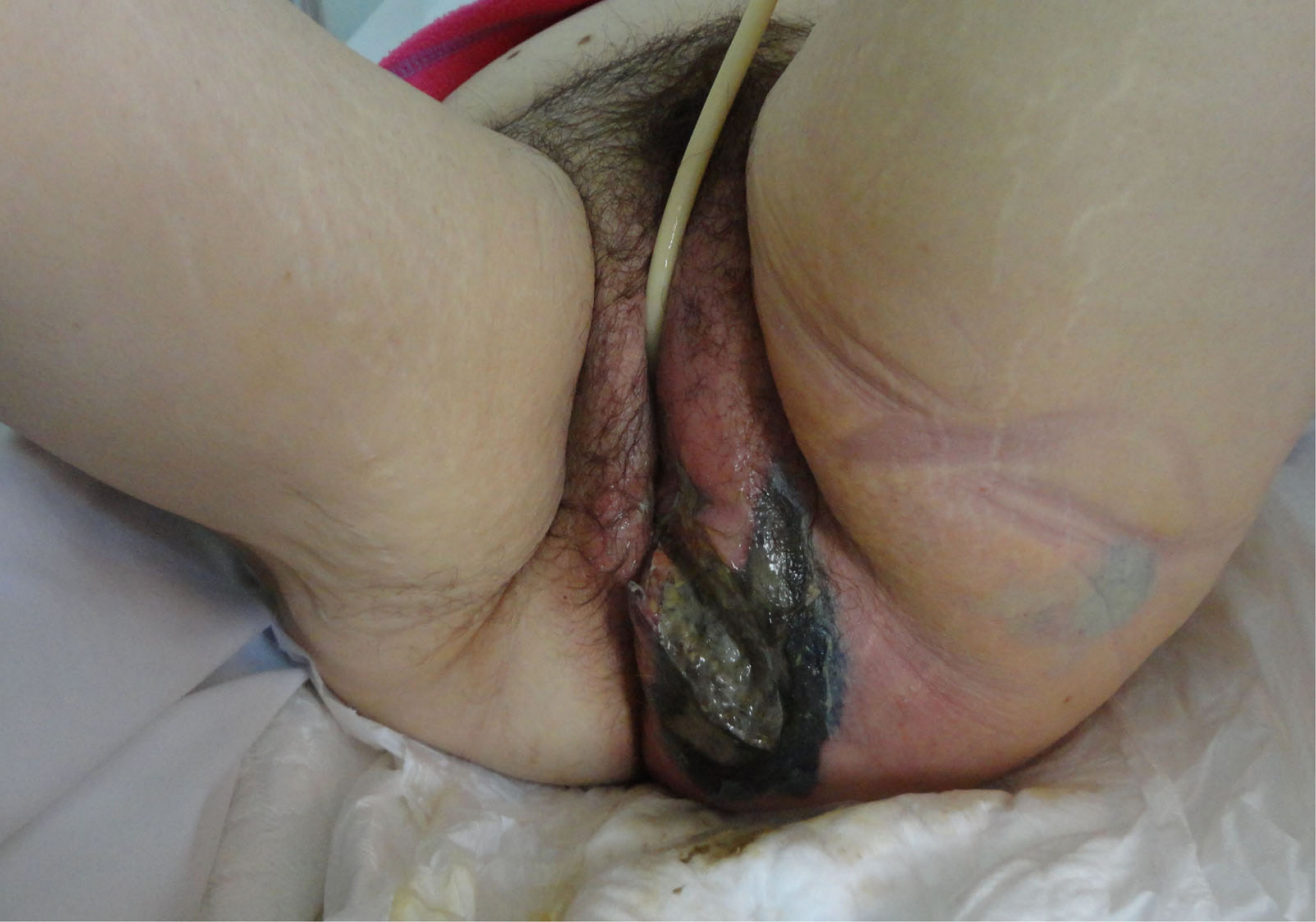
**A 65-year-old woman with extensive gas gangrene at the perineal area was managed with extensive debridement of necrotic skin and subcutaneous tissue**.

From 2005 to 2015, 62 patients were admitted to the 4 centers collaborating in the study with a diagnosis of νF. The mean age of the patients was 63.7 (median: 56 years old, range 37–87 years) with a male-to-female ratio of 3:1. Fournier’s gangrene was the predominant type of NF, as the perineum was infected in 29 patients (46.8%) (Figure 1). Twenty-two patients had infection of the lower limbs (35.5%). The upper limbs’ axillary region was infected in only five patients (8.1%) and the infection was spread in the abdominal wall in nine patients (14.5%). Diabetes mellitus was the most common comorbidity, as 25 patients had deregulated diabetes mellitus (40.3%). Hypertension was also a relatively common comorbidity (25.8%), as well as obesity (17.74%). Other comorbidities recorded were liver cirrhosis, renal impairment, and chronic heart failure (Table 1).

**Table 1:** **Patients’ characteristics**.

	Number of patients	Percentage	Correlation with mortality (*P* value)
**Demographics**			
Age, mean	63.7		<0.01
Female gender	15	24.2	0.04
**Site of infection**			
Perineum	29	46.8	
Lower limbs	22	35.5	
Upper limbs	5	8.1	
**Comorbidities**			
Diabetes mellitus	25	40.3	0.10
Hypertension	16	25.8	0.14
Obesity	11	17.7	0.33
Liver cirrhosis	3	4.8	0.09
Renal impairment	2	3.2	0.08
Chronic heart failure	2	3.2	0.08

The univariate analysis demonstrated that advanced age (over 65 years), with *P* value <0.01, as well as female sex (*P* = 0.04), was statistically significantly correlated with mortality. Univariate analysis of patients’ comorbidities showed no statistically significant correlation with mortality. Although not statistically significant, our results are indicative for a correlation between renal impairment and chronic heart failure and mortality (*P* = 0.08).

Nearly all patients were admitted in a serious condition, with both systemic and local symptoms. The vast majority had tenderness (90.3%) and pain (77.4%) on the infected site, which in some cases was inexorable. In 46 patients, the site of infection was edematous (74.2%), and in 43 patients, the infected skin was erythematous (69.4%). However, the simultaneous presence of these 3 symptoms, pain, tenderness, and erythema, which are characterized in the literature as “the classic triad of NF,” was recorded in only 16 patients (25.8%). In addition, the infected skin was found necrotic in 29 patients (46.8%), and in 14 patients hemorrhagic bullas had developed (22.6%). Crepitus was present in six patients (9.7%), manifesting gas gangrene. As far as the systematic symptoms are concerned, tachycardia was found in 21 patients (33.9%) and 19 patients were febrile (30.7%). Nine patients (14.5%) had developed hypotension (systolic blood pressure <100 mmHg) and 8 of them finally developed septic shock (12.9%), which was strongly correlated with mortality (*P* < 0.01) (Table 2).

**Table 2:** **Patients’ symptomatology**.

	Number of patients	Percentage
**Local symptoms**		
Tenderness	56	90.3
Pain	48	77.4
Swelling	46	74.2
Erythema	43	69.4
Necrotic skin	29	46.8
Hemorrhagic bullas	14	22.6
Crepitus	6	9.7
**Systemic symptoms**		
Tachycardia (<10 bpm)	21	33.9
Fever (38°C)	19	30.7
Hypotension (SAP <100 mmHg)	9	14.5
Septic shock (at least 2 criteria)	8	12.9

The retrospective evaluation of laboratory tests provided interesting results. The mean white-blood-cell count was 16,008/mm^3^ (median 15,476, 8,450–32,520), and in 45 patients WBC was over 15,000/mm^3^ (72.6%). C-reactive protein was elevated in 39 patients (62.9%), and its mean value was 195 mg/L (median 193, range 112–282). Anemia (hemoglobin <13.5 d/dL) was found in 47 patients (75.8%), but hemoglobin levels under 11 mg/dL were found only in 12 patients (19.4%). Mean serum sodium levels were calculated at 132.65 mmol/L (median 131, range 125–135) and mean creatinine levels at 153 mmol/L (median 150, range 90–210). Mean blood glucose value was 202.35 (11.242 mmol/L), with a median value 196 g/dL. Using the results of the above laboratory values in every patient, we retrospectively calculated the LRINEC score. Interestingly, 26 patients had LRINEC score under 6, which is regarded as the cutoff value in terms of NF severity (41.9%). Twenty patients had LRINEC score 6–8 (32.6%), and 16 patients had LRINEC score over 9 (25.8%). In terms of imaging studies, an X-ray was done in the vast majority of patients (50 patients; 80.6%), mainly for excluding other possible diseases. CT of the infected site was performed in 19 cases, in which the diagnosis was not yet ascertained (30.7%). CT findings were mainly fascial swelling, inflammation, and gas formation; furthermore, CT was helpful in showing the extent of tissue infection.

The majority of patients were initially treated with aggressive fluid resuscitation and empirical antibiotic treatment consisted of ampicillin–sulbactam and metronidazole. In 11 cases (17.7%), a carbapenem, mainly meropenem was used instead, with no significant difference regarding the patient’s clinical condition. The results of culture’s sample were not available for all patients; therefore, the pathogens were not recorded. Antibiotic treatment was started in almost all patients intraoperatively, and it was continued proportionally to infection parameters. The mean duration of antibiotic treatment was 18.2 days (median 13; range 1–41 days). The average time from admission to operation was 12.8 h (median 8.7 h, range 5.3–27.2 h), and only in three cases the surgery was delayed more than 24 h (4.8%). We performed aggressive surgical debridement in all patients, and we performed repeated debridement in almost all of them. The median number of repetitive debridement needed for every patient was 5 (1–9). In eight cases with infection of the abdominal wall, the underlying peritoneum was infected, and part of the underlying large intestine was not viable; therefore, a colectomy was required (12.9%). Moreover, in five patients with Fournier’s gangrene, an orchiectomy was also performed (8.1%). Furthermore, in 16 cases (25.8%) with NF of the upper and lower limbs, we proceeded to the amputation of the infected limb (5 upper limb amputations–11 lower limb amputations). Thirty-two patients were transferred immediately after operation to the intensive care unit (ICU) (51.6%). The mean length of ICU hospitalization was 2.45 days; however, the median length of ICU hospitalization was 12 days (0.75–35). In four patients, we used a vacuum-assisted device to accelerate wound healing. This technique had excellent results in terms of wound healing and tissue viability but did not reduce significantly the hospitalization period (*P* = 0.20). The mean length of hospital stay was 19.7 days with a median length of stay of 14 (10–43), and the overall mortality rate of our series was 17. 7% (Table 3).

**Table 3:** **Surgical treatment and outcome**.

	Number of patients	Percentage
**Surgical treatment**		
Median no of debridements	4.8	
Limb amputation	16	25.8
Colectomy	8	12.9
Orchiectomy	5	8.1
ICU admission	32	51.6
Mortality	11	17.7

## Discussion

Necrotizing fasciitis is a rare clinical entity, with an annual incidence of 1,000 cases annually, and global prevalence of 0.040 cases per 1,000 person-years (10). Numerous studies have shown that there is a preference for men, with a male-to-female ratio of 3:1; this ratio results most possibly from the increased incidence of Fournier’s gangrene in men (11). There is no age predilection for NF; however, middle-aged and elderly patients (over 50 years of age) are more likely to be infected (2). Indeed, the median age of our patients is comparable to other clinical series (12). The advanced age of patients with NF seems to be a crucial risk factor for higher mortality. We noted a statistically significant correlation between advanced age and mortality, and this is in accordance with large clinical studies that have shown that advanced age is a strong, independent predictor of mortality (13). However, other studies have maintained that although advanced age is a risk factor for mortality, it must be accompanied by a more aggressive clinical course as well (14). Unlike age, sex as a risk factor for mortality is still a topic of debate. In our series, there was a correlation between female sex and mortality, with a considerable level of significance. Moreover, in a study from Czymek et al., the mortality was significantly higher among females (50% F vs. 7.7% M) (15), but in larger clinical studies, female sex did not seem to affect mortality (16). As far as the site of infection is concerned, in a large clinical study from Anaya et al., lower extremities were mostly infected (57, 8%), followed by the abdomen and the perineum (17), unlike our series in which Fournier’s gangrene was the predominant form. Nevertheless, it is widely agreed that NF of the upper limbs is significantly rare compared to that of the lower limbs (18).

Patients with NF, mainly due to their advanced age, usually have at least one comorbidity. The most frequent comorbidity is diabetes mellitus. Goh et al. calculated the prevalence of diabetes mellitus in patients with NF at 44.5%, which is quite close to our series (12); it is doubtful whether diabetes mellitus affects mortality (5, 18). In our series, there was no statistically significant correlation between diabetes and mortality. Chronic renal failure is also a frequent comorbidity in patients with NF, which seems to be a decisive risk factor for mortality. Elevated serum creatinine, along with elevated blood urea, is strongly associated with high mortality rates (19). Other common comorbidities include preexisting hypertension, obesity, liver cirrhosis, chronic heart failure, alcohol abuse, immunodeficiency, systemic lupus erythematosus, Addison’s disease, and peripheral vascular disease (6, 7).

The clinical onset of patients with NF is not always evident, leading usually to misdiagnosis. The most common symptoms are local pain, swelling, and erythema; however, the simultaneous presence of these three symptoms is not a common phenomenon (20). Local skin changes consist of tenderness, crepitus, skin necrosis, and hemorrhagic bullas. The presence of crepitus suggests infection from anaerobic bacteria, which is useful for treatment strategy. Regularly, these symptoms combine with tachycardia (>100 beats/min) and fever, followed by hypotension (SAP <100 mmHg) and tachypnea (>20/min). According to the ACCP/SCCM Consensus Conference Committee, the presence of two or more from the above (or 1 and WBC >12 × 10^3^/mm^3^ or WBC <4 × 10^3^/mm^3^) indicates development of systemic inflammatory response syndrome (21). Taking into account that NF is caused by bacteria/fungi infection, we assume that patients who meet the SIRS criteria can develop sepsis, and when hypotension is resistive to fluid resuscitation patients may develop septic shock, which is indisputably a risk factor for mortality. In our series, all patients who developed septic shock did not manage to survive (*P* < 0.01). This strong correlation between septic shock and mortality has been repeatedly reported in the literature (22–24).

As the clinical presentation of patients with NF is not characteristic, the laboratory tests can provide not only useful information regarding the diagnosis of NF but may also indicate its severity. Elevated white-blood-cell count is a common feature in patients with NF (25) and white-blood-cell count in excess of 20,000/L is usually present in this disease. Blood urea nitrogen >18 mg/dL and serum creatinine >1.2 mg/dL may also be present, implying ongoing renal failure, which is often present in these patients. It is also suggested that C-reactive protein >16 mg/dL or creatine kinase >600 IU/L generally precludes group A b-hemolytic streptococcal infection (26). Apart from these observations, the severity of NF can be generally estimated utilizing the Laboratory Risk Indicator for NF (LRINEC) (27). After numerous studies for validation of LRINEC, it has been proposed that the cutoff value for diagnosis of NF is 6 and the severity of the infection can be estimated as follows: low (score <6), moderate (<8), and severe (≥9). In our series, 46 patients had LRINEC score under 8 (26 of them under 6), which indicates that the two-thirds of study population had a moderate form of NF. This can indirectly explain many of the results of our treatment, like relatively low hospital rate, and foremost our low mortality rate. However, in most of our cases, the diagnosis was equivocal; therefore, we used CT more frequently than in other series to set the diagnosis (16). Apart from CT and plain radiography, which we widely used, magnetic resonance imaging (MRI) or ultrasonography can also be used. Plain radiography is useful only in cases of gas gangrene, where gas formation is present. Despite its low sensitivity, it is generally widely used due to its low cost (28). A CT scan can demonstrate the extent of tissue infection, fascial swelling, inflammation, and gas formation. An MRI scan may provide additional information but is rarely used. Ultrasonography is also a feasible option, mainly in cases of gas gangrene. Bedside tests such as finger test and frozen section biopsy are occasionally used for confirmation of diagnosis, and when the diagnosis remains unclear, surgical exploration can set the diagnosis (29). Common findings in these bedside tests is the characteristic “dishwater pus,” along with the lack of bleeding and lack of tissue resistance to blunt finger dissection (20). Actually, the combination of surgical exploration and microbiological and histopathological analysis of 1 cm^3^ of soft tissue is considered the gold standard for confirming diagnosis, when the latter is ambivalent; however, because of the acute nature of the disease this combination is rarely used by the clinicians.

Undoubtedly, the time from admission to surgery is the most decisive factor for survival. Emergency surgical debridement should be performed in all patients within 12–15 h after admission, since a delay in treatment beyond 12 h especially in the fulminant forms of NF can prove fatal (30). At any case a delay over 24 h is unacceptable, as the mortality rate can be nine times greater when primary surgery is performed 24 h after the onset of symptoms (4). We managed to maintain a mean time of 12.8 h from admission to surgery, which eventually contributed to higher survival rates. Nonetheless, before surgery and during diagnostic procedures, patients should be resuscitated with crystalloids, and broad-spectrum antibiotics should be given. Although blood culture results are not always available in an emergency basis, the empirical usage of antibiotics is based on the suspected microbiological type of NF. Medical history and imaging tests can also be indicative for the microbiological type. When a polymicrobial infection is suspected, ampicillin or ampicillin–sulbactam combined with metronidazole or clindamycin are used (31). Alternatively, carbapenems can be administered. In cases of previously hospitalized patients, piperacillin–tazobactam, ticarcillin–clavulanate acid, third- or fourth-generation cephalosporins, or carbapenems are used, but at a higher dosage. Monomicrobial infection by beta-hemolytic streptococcus A is treated with first- or second-generation cephalosporins, except of cases with suspected MRSA coinfection, in which vancomycin, or daptomycin and linezolid are used (31). Gas gangrene is usually a result of *Clostridium* species infection and is treated with clindamycin and penicillin. Finally, NF caused by fungi can be treated with amphotericin B or fluoconazole, with disappointing results (20).

**Figure 2:**
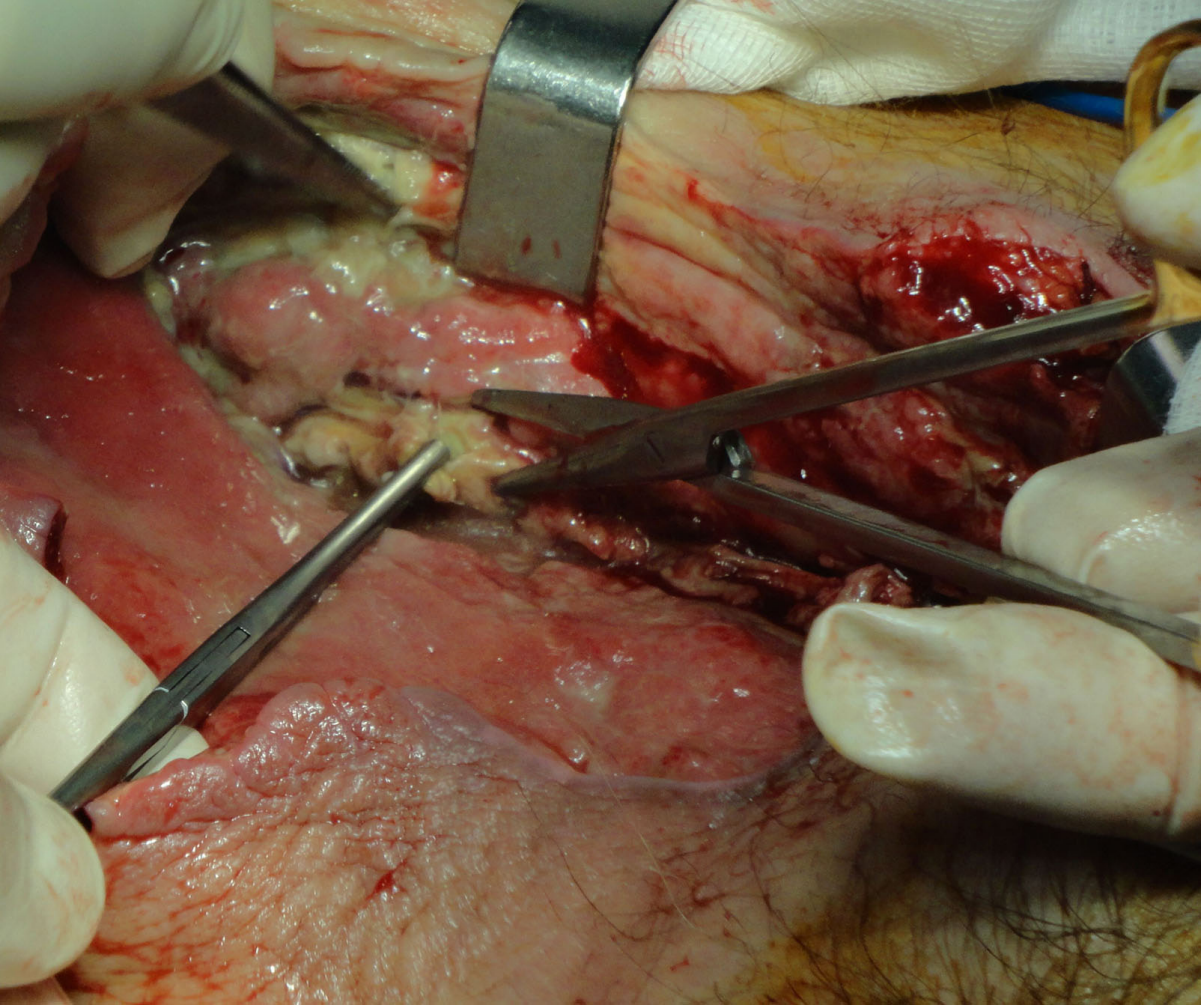
**A 75-year-old woman with gas gangrene at the proximal right thigh**. Aggressive surgical debridement with excision of all necrotic tissues was performed.

Aggressive surgical debridement, necrosectomy, and fasciotomy are the main points of surgical treatment. Barely one surgical debridement is enough for proper treatment. Usually, debridement is repeated during the next 24 h or later, depending on the clinical course and patient’s general condition. Special consideration is required for the extent of the first debridement. Generally, debridement should be extended until healthy tissue is found (32) (Figure 2). In a study from Mok et al. the relative risk of death was 7.5 times greater when a restricted primary debridement was performed (32). When the surgical wound after the first debridement is considered as complicated, a “second-look operation” with radical surgical debridement is usually required (33). In addition, it has been reported that a complicated surgical wound may require up to 40 additional operations (10). Our mean number of debridement repetitions was 4.8, which are relatively high compared to other series (12), but we strongly believe that this contributed a lot to our low mortality rate. In NF of the limbs, there is always a dilemma on amputation of the infected limb. Tang et al. have proposed specific criteria for amputation, and the most significant is extensive soft tissue necrosis with involvement of the underlying muscles and rapidly progressing infection with a large area of tissue necrosis (34). Amputation is associated with less blood loss than a radical debridement; therefore, patients with fulminant form of NF who have already developed septic shock are clearly benefited (20). Notwithstanding, limb amputations overall do not affect significantly the mortality rate (5).

Postoperative treatment is also crucial for survival. Any evidence of hemodynamic instability demands immediate resuscitation, transfer to an ICU, nutritional support, and enteral feeding. The majority of our patients were transferred to ICU, although the mean length of stay in ICU was relatively short. As far as the wound healing is concerned, we observed a significant reduction of wound surface area in cases where VAC devices were used. However, there was no statistical significant difference in terms of the hospitalization period (*P* = 0.20), and combining the higher cost of VAC therapy compared to conventional gauze therapy, we suggest VAC be used only in wounds with large surface and/or in patients with several comorbidities.

The mortality rate of our series was 17.7%, which is considerably low compared to other series (12). Overall, the mortality rate of patients with NF is a controversial issue. Goh et al. concluded that a median mortality ratio was 21.5% (12), but mortality rates from 8.7 to 76% have been reported (35). Nonetheless, without treatment, the mortality rate of this disease reaches 100% (20). Therefore, early diagnosis and early surgical treatment are the key points in managing this disease. More frequently, using imaging studies, we managed to maintain a low time period from admission to surgery, which indisputably played a positive role in terms of survival. The mean LRINEC score of patients showed that the severity of the majority of our cases was moderate, which can explain our low mortality rate. Apart from that, we need to point that repeated extensive surgical debridement undoubtedly leads to better outcomes regarding mortality. Moreover, the repetitive use of surgical debridement compared to other studies played a significant role in better survival compared to other studies. Finally, hospitalization of the patients in ICU for a more intensive postoperative treatment was also compelling.

## Ethics Statement

This study been accomplished after it had taken approval by the Bioethics Committee of Attikon University Hospital numbered EBD 1578/2-6-2016 at the 16th meeting of 6-7-2016.

## Author Contributions

EM: study design, study supervision, and discussion section. GB: data collection, methodology, and results section. IP: clinical advisor, results section, and study supervision. ND: data collection, methodology, and results section. PP: clinical section and results section. NM: data collection, methodology, and results section. TK: data collection, results section, and discussion section. NArkadopoulos: study design and discussion section. KT: data collection, methodology, and results section. NAlexakis: data collection, methodology, and results section. MK: study supervision and discussion section. GZ: clinical section, results section, and discussion section. VS: clinical section and discussion section. GK: study design, data interpretation, and discussion section. AM: clinical section, results section, and discussion section.

## Conflict of Interest Statement

The authors declare that the research was conducted in the absence of any commercial or financial relationships that could be construed as a potential conflict of interest. The reviewer RA and handling Editor declared their shared affiliation, and the handling Editor states that the process nevertheless met the standards of a fair and objective review.
